# Potential Anxiolytic Effects of Selected Inositol Stereoisomers—A Narrative Review

**DOI:** 10.3390/cells15110970

**Published:** 2026-05-24

**Authors:** Maria Derkaczew, Kamila Zglejc-Waszak, Piotr Podlasz, Marcin Jozwik, Joanna Wojtkiewicz

**Affiliations:** 1 Department of Human Physiology and Pathophysiology, School of Medicine, Collegium Medicum, University of Warmia and Mazury, 10-082 Olsztyn, Poland; mariaderkaczew@gmail.com; 2 Department of Anatomy and Histology, School of Medicine, Collegium Medicum, University of Warmia and Mazury, 10-082 Olsztyn, Poland; kamila.zglejc@uwm.edu.pl; 3 Department of Pathophysiology, Forensic Veterinary Medicine and Administration, Faculty of Veterinary Medicine, University of Warmia and Mazury, 10-719 Olsztyn, Poland; piotr.podlasz@uwm.edu.pl; 4 Department of Gynecology and Obstetrics, Collegium Medicum, University of Warmia and Mazury, 10-045 Olsztyn, Poland; marcin.jozwik@uwm.edu.pl

**Keywords:** anxiety, inositol, myo-inositol, scyllo-inositol, d-chiro-inositol

## Abstract

**Highlights:**

**What are the main findings?**

- Available preclinical and experimental studies suggest a biologically plausible anxiolytic effect of selected inositol stereoisomers, mainly myo-inositol, scyllo-inositol, and D-chiro-inositol.
- Evidence from human studies remains limited, heterogeneous, and largely indirect, with a lack of well-designed, anxiety-focused clinical trials.

**What are the implications of the main findings?**

- Inositol stereoisomers represent promising but still exploratory candidates for anxiety modulation via intracellular signaling and neurotransmission pathways.
- Future research should prioritize standardized anxiety outcomes, dose optimization, and adequately powered translational and clinical studies.

**Abstract:**

Background: Anxiety is a frequent clinical problem that becomes disabling when excessive or persistent. Cyclitols are naturally occurring polyhydroxy compounds, and inositols are the most abundant cyclitols in eukaryotic cells; several stereoisomers have been proposed as candidates for CNS-relevant effects. Methods: A narrative review was conducted using a structured search of biomedical bibliographic databases. The search was centered on myo-inositol, scyllo-inositol, and D-chiro-inositol in relation to anxiety-related outcomes. Results: The retrieved literature suggests some biological plausibility for anxiolytic effects of selected inositol stereoisomers through pathways related to intracellular signaling and neurotransmission. However, the available evidence is uneven and remains limited. The most informative findings concern myo-inositol and include both preclinical and clinical studies, whereas data on scyllo-inositol and D-chiro-inositol are scarce, particularly in relation to anxiety-related outcomes. Conclusions: Current evidence suggests a possible anxiolytic role of selected inositol stereoisomers; however, the existing data are limited and heterogeneous, and do not allow for definitive clinical conclusions. Further research is required.

## 1. Introduction

Anxiety plays a dual role, encompassing both adaptive and maladaptive dimensions. As an evolutionarily conserved response to threat, it is essential for survival by promoting vigilance and facilitating appropriate defensive behaviors. However, when anxiety becomes excessive, disproportionate, or persistent beyond what is necessary for safety, it can markedly impair daily functioning and overall quality of life. Anxiety can manifest in many different clinical contexts and is not limited to psychiatric illness. In routine practice, it may accompany somatic conditions, for example, endocrine dysregulation or cardiovascular disease, and it can also be observed in substance use disorders, either during intoxication or as part of withdrawal. Anxiety symptoms may also be precipitated or exacerbated by exposure to certain medications, including agents with central nervous system effects [1,2]. When anxiety becomes the primary clinical phenomenon rather than a secondary symptom, it defines the spectrum of anxiety disorders, including generalized anxiety disorder, social anxiety disorder, panic disorder (with or without agoraphobia), and specific phobias. These disorders typically involve persistent apprehension and excessive worry, fear related to social evaluation or performance, recurrent panic episodes that may be unexpected or situation-linked, heightened anticipatory tension, and avoidance strategies. Importantly, anxiety disorders frequently present with prominent physical complaints such as palpitations, dyspnea, and dizziness, which contribute to their high visibility in primary care [3]. Since the emergence of SARS-CoV-2 in late 2019, the COVID-19 pandemic has been linked to a substantial increase in anxiety and depressive symptoms worldwide [4]. Anxiety and depressive disorders frequently co-occur, and converging evidence from symptom profiles, longitudinal course, shared neurobiological markers, familial aggregation, and treatment response supports a substantial overlap between these conditions [1].

Despite the broad range of available psychopharmacological options, the treatment of anxiety and depressive disorders remains clinically demanding. In both conditions, selective serotonin reuptake inhibitors (SSRI) and serotonin-noradrenaline reuptake inhibitors (SNRI) are commonly recommended as first-line long-term therapies. In contrast, other antidepressant classes, including mirtazapine, bupropion, tricyclic antidepressants, and monoamine oxidase inhibitors, are used when initial strategies are ineffective or poorly tolerated. For anxiety in particular, benzodiazepines are still prescribed in selected situations because of their rapid symptomatic relief. Yet, their use is generally limited to short-term or adjunctive treatment due to sedation, cognitive and psychomotor impairment, tolerance, withdrawal phenomena, and dependence liability. In real-world settings, adverse effects, drug–drug interactions, delayed onset of benefit, and incomplete remission frequently lead to dose modifications, switching, or augmentation approaches, underscoring substantial interindividual variability in both efficacy and tolerability. Current pharmacological treatments for anxiety act through multiple, distinct mechanisms, ranging from GABA_A_ receptor modulation (e.g., benzodiazepines) to monoaminergic approaches (e.g., SSRIs/SNRIs), 5-HT1A partial agonism (buspirone), and neuromodulation of calcium channel signaling (pregabalin) [5,6,7,8,9,10]. Increasingly, this heterogeneity is linked to differences in pharmacokinetic and pharmacodynamic pathways, which has fueled interest in pharmacogenetic strategies aimed at moving beyond conventional trial-and-error prescribing; large pragmatic studies suggest that genotype-guided treatment can reduce clinically relevant adverse drug reactions [11]. Importantly, even agents considered comparatively “benign”, such as non-benzodiazepine anxiolytics, may rarely cause significant neurological adverse effects in vulnerable individuals, as illustrated by reports of buspirone-associated dyskinesia or dystonia, plausibly related to its interactions with dopaminergic signaling [12]. Taken together, these limitations and safety considerations strengthen the rationale for investigating safer, well-tolerated alternatives or adjuncts with mechanistic novelty, including compounds that may influence intracellular signaling pathways relevant to mood and anxiety regulation.

**Figure 1:**
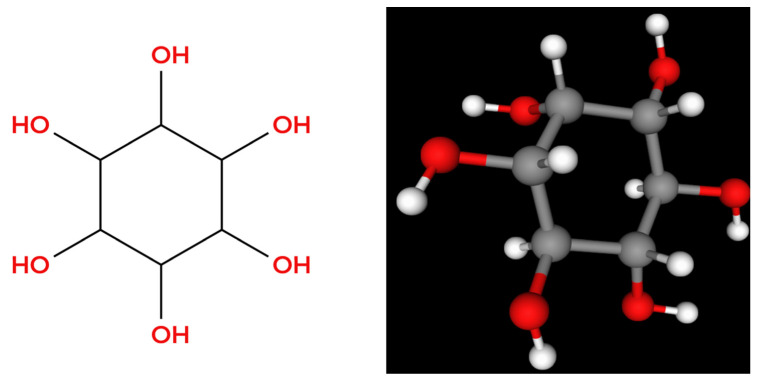
Myo-inositol (MI) configuration. Figure was created in MolView (https://molview.org, accessed on 16 April 2026).

Cyclitols constitute a broadly distributed class of naturally occurring polyhydroxy compounds, with myo-inositol and related molecules among the most prominent representatives. Owing to their involvement in multiple metabolic and signaling pathways, cyclitols have attracted increasing attention for a wide spectrum of biological effects [13]. Inositol is an integral component of the intracellular phosphatidylinositol second messenger system, which interacts with multiple neurotransmitter receptors, including those for serotonin, dopamine, and glutamate, thereby influencing central neurotransmission, particularly serotonergic signaling [14]. Recent reports have also raised the possibility that selected cyclitols may exert anxiolytic activity; however, robust clinical evidence remains scarce, and the available data are largely preliminary, often derived from indirect observations or preclinical research [13]. Structurally, cyclitols are cycloalkane-based polyols characterized by a ring scaffold bearing at least three hydroxyl substituents, each located on a different carbon atom. In eukaryotic cells, inositols represent the most abundant and biologically relevant cyclitols. Notably, nine stereoisomeric forms of inositol have been described: myo-inositol (Figure 1), scyllo-inositol, D-chiro-inositol, L-chiro-inositol, cis-inositol, epi-inositol, muco-inositol, allo-inositol, and neo-inositol. In this narrative review, we focus on three inositol stereoisomers, myo-inositol, scyllo-inositol, and D-chiro-inositol, because they are the most biologically and clinically relevant forms in humans and, importantly, the ones for which mechanistic rationale and the largest body of experimental literature are currently available. At the same time, for all three compounds, direct, well-powered clinical evidence for anxiolytic efficacy remains limited, and consistent statistically significant effects on anxiety or depressive symptoms have not yet been firmly demonstrated [15,16,17].

## 2. Materials and Methods

This narrative review summarizes and critically discusses the available evidence on the potential anxiolytic effects of selected inositol stereoisomers, with a primary focus on myo-inositol, scyllo-inositol, and D-chiro-inositol. A literature search was performed in PubMed supplemented by targeted Google Scholar queries. The final search was performed on 18 April 2026, and the search process covered publications available up to that date. Search terms combined inositol-related keywords (e.g., “cyclitols”, “inositol”, “myo-inositol”, “scyllo-inositol”, “D-chiro-inositol”) with anxiety- and stress-related outcomes (e.g., “anxiety”, “anxiolytic”, “stress”, “panic”, “depression”). Searches were performed using different combinations of these keywords to maximize the retrieval of potentially relevant studies. We included original preclinical and clinical studies reporting anxiety-relevant behavioral, clinical, neurochemical, or mechanistic outcomes following exposure to the target stereoisomers, as well as translational papers supporting biological plausibility; narrative and systematic reviews were screened primarily to identify additional primary sources. Studies were excluded if they did not involve the selected stereoisomers, did not report anxiety-related endpoints, or were insufficiently informative for the scope of this review. Evidence was prioritized according to its relevance to the review question and its methodological informativeness. Greater weight was given to studies directly assessing anxiety-related endpoints, studies with explicit intervention characteristics, and reports providing interpretable outcome measures and comparator conditions. Human studies were considered particularly relevant for translational interpretation, whereas animal and mechanistic studies were used to support biological plausibility and hypothesis generation. Because of substantial heterogeneity across experimental models, study populations, dosing regimens, routes of administration, treatment durations, and outcome measures, the findings were synthesized qualitatively rather than quantitatively. The main extracted variables included study design, model or population, intervention characteristics (compound, dose, route, and duration), comparator conditions, outcome measures, principal findings, and reported safety or tolerability signals.

## 3. Inositols

### 3.1. Myo-Inositol

MI is a ubiquitous carbohydrate that is highly abundant in the central nervous system, where it plays a key role as a secondary messenger in neuronal signaling, membrane turnover, and osmoregulation. It constitutes a core component of the inositol signaling system, acting as a precursor of membrane phosphatidylinositols and inositol phosphates, including inositol trisphosphate, which function as second messengers in numerous neurotransmitter receptor pathways such as serotonergic, adrenergic, muscarinic, glutamatergic, and histaminergic signaling, as well as other neuromodulatory systems relevant to central nervous system function and stress regulation [18,19]. MI is obtained both from the diet, predominantly from plant-based foods including cereals, nuts, and fruits, and through endogenous synthesis from glucose-6-phosphate. Endogenous production occurs mainly in the kidney and liver via a two-step enzymatic pathway involving myo-inositol-1-phosphate synthase and inositol monophosphatase, which likely contributes to the absence of clearly defined dietary intake requirements in humans and to marked tissue-specific variability in MI levels. The kidney also serves as the primary site of MI metabolism and catabolism, where it is oxidized to D-glucuronic acid and further processed through the glucuronate–xylulose pathway into D-xylulose-5-phosphate, an intermediate of the pentose phosphate pathway. This pathway is essential for maintaining cellular homeostasis, supporting redox balance via NADPH production, and providing ribose-5-phosphate for nucleotide synthesis, thereby linking MI metabolism to fundamental cellular and metabolic processes [19]. MI pathway activation initiates intracellular signal transduction through a cascade of interacting proteins, including GTP-binding Gq proteins (Gαq/11/14/15/16 family), which subsequently stimulate membrane-associated phospholipase C. This leads to the generation of inositol 1,4,5-trisphosphate, which diffuses into the cytosol and triggers the release of calcium from the endoplasmic reticulum, thereby activating multiple calcium-dependent enzymes and receptors. As a key component of the phosphatidylinositol pathway, MI contributes to the generation of inositol trisphosphate and diacylglycerol, which regulate calcium-dependent signaling, synaptic activity, and neuronal excitability. Through these mechanisms, MI influences neurotransmitter systems implicated in anxiety and mood regulation, including serotonergic, dopaminergic, and gamma-aminobutyric acid-related pathways, and supports osmotic balance and cellular homeostasis under stress conditions. Within the central nervous system, MI required for this signaling pathway is supplied through receptor-driven recycling mechanisms, endogenous de novo synthesis, and dietary intake [17,20]. Preclinical and clinical evidence indicate that myo-inositol exerts psychoactive properties, with anxiolytic and antidepressant effects reported in several experimental models and human studies [21,22,23,24,25,26,27]. Randomized controlled trials have demonstrated that myo-inositol supplementation can reduce the frequency and severity of panic attacks, with efficacy comparable to selective serotonin reuptake inhibitors in some settings. At the same time, its effects on other anxiety dimensions appear more limited. These clinical observations are consistent with a mechanism of action that differs from conventional pharmacotherapy. MI exists in a free form and acts as a precursor to intracellular second messengers. MI modulates intracellular signaling cascades rather than directly targeting neurotransmitter reuptake or receptor binding [17].

The first clinical investigations into the putative anxiolytic effects of inositol, primarily focusing on MI as the most abundant naturally occurring stereoisomer, were initiated in 1995. This line of research was motivated by earlier reports suggesting potential benefits of inositol in mood disorders, which prompted an evaluation of its impact on anxiety-related symptoms, including panic attacks. In a randomized, placebo-controlled trial, Benjamin et al. reported that inositol administration was associated with a significantly greater reduction in both the frequency and severity of panic attacks, as well as decreased agoraphobia severity, compared with placebo, with minimal adverse effects observed. The authors noted that the combination of apparent clinical efficacy, favorable tolerability, and the fact that inositol is a naturally occurring component of the human diet supports its consideration as a potentially attractive therapeutic option for panic disorder [16,22]. A randomized controlled trial conducted in 1996 evaluated high-dose inositol supplementation in a small cohort of patients with post-traumatic stress disorder. Four weeks of treatment at a daily dose of 12 g did not result in measurable improvement in core PTSD symptoms, including avoidance and intrusive phenomena, although a modest reduction in depressive symptoms was observed in a limited subset of participants [28]. Another work from 1997 proposed that altered availability of inositol within the central nervous system may disrupt intracellular signaling pathways and thereby contribute to the development of neuropsychiatric disorders. Initial clinical observations suggested that MI may exert psychoactive effects, with reported benefits in conditions such as depression, panic disorder, and obsessive–compulsive disorder, supporting the concept that modulation of post-receptor second messenger cascades, rather than direct receptor targeting, could represent a novel therapeutic strategy in psychiatry [18]. A double-blind, randomized crossover trial in 2001 compared MI with fluvoxamine in patients with panic disorder, providing a direct comparison with an established pharmacological treatment. Over two consecutive one-month treatment periods, MI administered at doses up to 18 g/day produced improvements in anxiety severity, agoraphobia, and global clinical status comparable to those observed with fluvoxamine. Notably, during the first treatment phase, MI was associated with a greater reduction in the weekly frequency of panic attacks than fluvoxamine, and it also demonstrated a more favorable tolerability profile, with fewer gastrointestinal and fatigue-related adverse effects. These findings suggest that MI may offer a clinically relevant, well-tolerated alternative for panic disorder, although the need for replication in larger trials remains [21]. A double-blind, placebo-controlled randomized clinical trial published in 2017 evaluated the efficacy and tolerability of inositol (the specific inositol stereoisomer administered was not explicitly specified in the study protocol) in adults with trichotillomania, a compulsive disorder characterized by repetitive hair pulling. Over a 10-week treatment period, participants received inositol at doses ranging from 6 to 18 g/day and were assessed using validated measures of symptom severity, global clinical improvement, anxiety, and depressive symptoms. Inositol treatment did not result in significantly greater reductions in primary or secondary outcome measures compared with placebo. However, a numerically higher proportion of individuals in the inositol group were rated as much or very much improved at study completion. Overall, these findings indicate that inositol does not exert robust therapeutic effects in trichotillomania at the group level, highlighting that its clinical benefits may be limited to specific anxiety-related phenotypes, such as panic disorder, rather than extending broadly to compulsive or impulse-control disorders [29]. Zulfarina et al., in an article from 2019, proposed incorporating MI into Malaysian guidance for the management of panic disorder, to be considered alongside established pharmacological options and, in selected situations, potentially also as monotherapy. In the same context, the authors summarized positive preliminary findings suggesting that several less commonly emphasized agents, including duloxetine, reboxetine, mirtazapine, nefazodone, risperidone, and inositol, may be clinically useful in patients with treatment-resistant panic disorder. They further reported that, across the studies reviewed, these medications were generally described as well tolerated compared with SSRIs and benzodiazepines, which was discussed as potentially relevant for patients characterized by ambivalence and heightened hypervigilance [16,30].

Metabolic and gynecological disorders such as obesity, diabetes, and polycystic ovary syndrome are increasingly recognized as being closely linked with psychological symptoms, particularly depression and anxiety. Because these conditions frequently affect women of reproductive age, interest has grown in therapeutic strategies that may improve both metabolic and psychiatric outcomes. In this context, myo-inositol appears particularly relevant, as it may help alleviate psychological symptoms in patients with PCOS and related metabolic disturbances, while offering a favorable safety profile, including during pregnancy [31]. Moreover, MI has been reported to exert calming and anxiolytic-like effects and may also contribute to the alleviation of symptoms associated with premenstrual syndrome. In the context of dietary supplementation, more recent studies show that effective daily doses typically range from approximately 500 to 4000 mg, depending on the clinical indication and whether myo-inositol is administered alone or in combination with other bioactive compounds [23,32,33]. MI has also been investigated in women with premenstrual dysphoric disorder (PMDD), a condition characterized by significant mood disturbances linked to the menstrual cycle. Clinical data suggest that MI supplementation may lead to improvements in symptom severity, including reductions in daily symptom burden and improvements in standardized psychiatric rating scales. These findings are supported by meta-analytic data indicating a trend toward greater efficacy of MI in depressive symptoms among patients with PMDD compared to placebo [14,34]. Earlier studies employed relatively high doses of myo-inositol; however, mild gastrointestinal adverse effects, including nausea, flatulence, and diarrhea, were reported at doses around 12 g/day. Consequently, later studies have tended to use lower dosing regimens to improve tolerability [35].

MI is generally considered safe for use during pregnancy and has been extensively studied in pregnant women, primarily regarding metabolic outcomes, with no major safety concerns reported at commonly used doses. MI is generally well tolerated, with adverse effects limited mainly to mild gastrointestinal symptoms, such as nausea, at higher doses [17]. Beyond its metabolic effects, several clinical studies have explored the potential influence of myo-inositol-based supplementation on maternal mental health, mood, and anxiety-related outcomes during pregnancy and the postpartum period. A retrospective cohort study in women with mild to moderate perinatal depression found that supplementation with myo-inositol combined with probiotics and trace elements from preconception through pregnancy was associated with reduced anxiety and depressive symptoms postpartum, improved quality of life, and fewer pregnancy-related complications. The intervention was also linked to more favorable fetal growth parameters and neonatal outcomes, suggesting broader benefits for maternal–fetal health beyond mood regulation [23]. In a double-blind randomized trial in women planning pregnancy, supplementation with MI combined with micronutrients and probiotics from preconception through pregnancy did not significantly reduce anxiety or depressive symptoms during pregnancy or the postpartum period compared with standard micronutrient supplementation. However, the intervention was associated with a small but statistically significant improvement in overall mental health functioning from preconception to six months after delivery [36]. A randomized controlled trial in pregnant women showed that myo-inositol supplementation was associated with a modest but significant improvement in overall sleep quality, including subjective sleep quality, sleep duration, and sleep efficiency, compared with placebo. These findings suggest that myo-inositol may influence sleep-related neurobiological processes during pregnancy, potentially reflecting broader effects on central nervous system regulation rather than direct anxiolytic activity [33].

### 3.2. Scyllo-Inositol

SCI has been investigated in several randomized controlled clinical trials under the development code ELND005, primarily in neurodegenerative and selected psychiatric conditions rather than in anxiety disorders. Based on information available from public clinical trial registries, six interventional studies evaluating ELND005 have reported results, most of which were conducted in patients with Alzheimer’s disease, including trials addressing cognitive outcomes, behavioral symptoms such as agitation and aggression, and long-term safety [37]. Additional studies assessed short-term safety in young adults with Down syndrome without dementia and explored SCI as an adjunctive maintenance treatment in bipolar I disorder. Among these trials, two were terminated early, with available registry data indicating tolerability concerns at higher doses, particularly gastrointestinal adverse effects, as contributing factors. As the detailed efficacy outcomes of the completed studies require further examination of the published trial reports, the existing evidence base allows only cautious conclusions regarding the clinical profile of SCI, while highlighting the need for closer analysis of completed RCTs before drawing inferences relevant to anxiety-related indications. In a randomized Phase 2 trial of individuals with mild to moderate Alzheimer’s disease, treatment with ELND005 was linked to a lower incidence of newly developing neuropsychiatric symptoms relative to placebo, with the strongest effects observed in patients at earlier stages of the disease. Reductions were most pronounced in affective manifestations, such as depressive and anxiety-related features, indicating that SCI may alter susceptibility to behavioral and emotional disturbances rather than acting as a direct symptomatic agent. While these observations suggest a role for SCI in modulating neuropsychiatric profiles in neurodegenerative disorders, their implications for primary anxiety disorders remain indirect and warrant further study [38].

### 3.3. D-Chiro-Inositol

DCI is a biologically active stereoisomer of inositol that, together with MI, participates in intracellular signaling processes central to metabolic and cellular regulation. Most clinical and mechanistic knowledge on DCI derives from studies in metabolic disorders, particularly polycystic ovary syndrome, where combined supplementation with myo-inositol and DCI has been shown to improve insulin sensitivity, endocrine balance, and ovulatory function, with a favorable safety and tolerability profile. These findings support the concept that DCI acts as a functional modulator of inositol-dependent signaling rather than as an inert metabolic byproduct. At the cellular level, DCI contributes to phosphatidylinositol-dependent second messenger pathways that regulate calcium signaling, synaptic activity, and intracellular homeostasis, processes that are also fundamental to central nervous system function. Although direct evidence for anxiolytic or antidepressant effects of DCI remains limited compared with MI, its involvement in pathways linked to serotonin, dopamine, and gamma-aminobutyric acid signaling suggests potential relevance for mood and stress regulation [19,39]. Importantly, DCI cannot be readily converted back to myo-inositol, raising questions about tissue-specific balance between inositol isomers and their long-term neurobiological effects. Current supplementation strategies should oscillate between MI and DCI ratios intended to reflect physiological plasma proportions, most commonly 40:1, which appears to optimize therapeutic outcomes in metabolic conditions. Whether similar ratios are relevant for neuropsychiatric indications remains unknown. Overall, while DCI shows promise as part of combined inositol-based interventions and shares key intracellular signaling roles with MI, further targeted studies are needed to clarify its independent effects on anxiety-related outcomes, optimal dosing, and its interaction with myo-inositol in the central nervous system [17].

According to data available from the ClinicalTrials.gov registry, although DCI has been evaluated in dozens of registered studies, the overwhelming majority address non-psychiatric conditions (especially metabolic and reproductive disorders), and there are no clearly registered RCTs using DCI as a primary intervention for anxiety or panic disorders in the clinical trial registry. Several clinical trials involving DCI have recently reached completion in 2024, including studies designed to assess symptoms of hormonal imbalance and associated mood swings. Although these trials registered mood-related outcomes as part of their assessments, results have not yet been posted or published.

### 3.4. Other Stereoisomers

**Figure 2:**
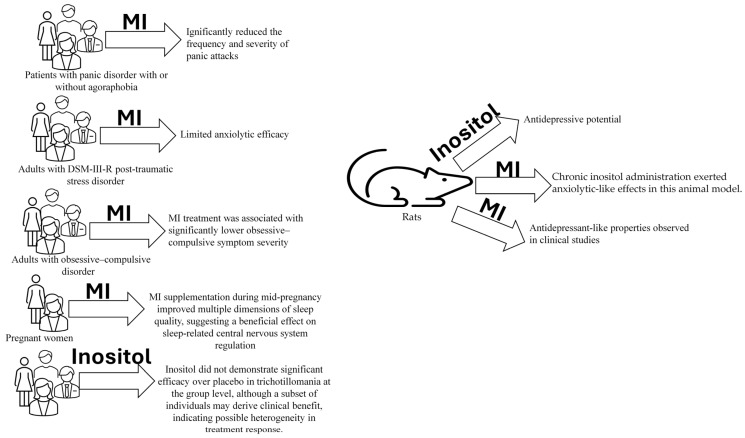
Summary of Table 1. Experimental and clinical studies. Selected inositol stereoisomers and their anxiolytic activity. Abbreviation: MI—myo-inositol.

Subsequent experimental work showed that epi-inositol (EI), an unnatural stereoisomer that does not serve as a substrate for phosphatidylinositol synthase, also exerts anxiolytic-like and anticonvulsant effects (Table 1, Figure 2). In a rat elevated plus maze model, chronic administration of EI reduced anxiety-like behaviors more effectively than MI, suggesting that inositol-related behavioral effects may involve additional mechanisms beyond classical phosphatidylinositol-dependent signaling, potentially related to differences in metabolism or alternative intracellular pathways. EI is a stereoisomer that does not naturally occur in mammalian tissues but has been identified in plant sources, including pine bark [24,39].

## 4. Alterations in Inositol Levels in Psychiatric Disorders

Growing evidence has indicated that MI levels are altered across a range of psychiatric conditions, including anxiety disorders, depression, and psychotic disorders. Both central measurements, primarily obtained using proton magnetic resonance spectroscopy (^1^H-MRS), and peripheral assessments in serum or plasma, suggest that these alterations may reflect underlying neurobiological dysregulation rather than condition-specific changes. However, the direction and magnitude of these differences appear to be region- and disorder-dependent [45]. An increase in MI observed on MRS may be associated with brain injury and glial-related pathological changes, elevated MI levels in the dorsolateral prefrontal cortex (DLPFC) have been described in individuals at clinical or genetic high risk for psychosis [16].

Evidence from ^1^H-MRS has highlighted region-specific alterations in MI concentrations within the anterior cingulate cortex (ACC) in association with anxiety-related symptoms. The study in preadolescent children found that exposure to traffic-related air pollution is associated with increased anxiety symptoms and measurable neurochemical alterations in the ACC, a brain region involved in emotional regulation. Proton magnetic resonance spectroscopy demonstrates that higher levels of air pollution exposure are accompanied by elevated MI concentrations in the ACC, which correlate with greater severity of generalized anxiety symptoms [46]. However, a magnetic resonance spectroscopy study in females with anorexia nervosa demonstrated reduced MI levels in the dorsal ACC compared with healthy controls, alongside markedly increased anxiety related to food intake. Notably, lower MI concentrations were associated with greater anxiety-to-eat in response to high-energy foods [47]. Similarly, reduced MI levels in the ACC have been reported in young individuals with unipolar depression and were linked to delayed melatonin onset, pointing to an association between altered myo-inositol–dependent signaling, anxiety-related symptoms, and broader neurobiological dysregulation rather than a uniform anxiolytic profile across conditions [48]. In addition, magnetic resonance spectroscopy data from patients undergoing electroconvulsive therapy (ECT) for severe depression demonstrated region-specific changes in MI over the course of treatment. MI levels increased in the dorsomedial ACC, whereas in the hippocampus, particularly on the left side, lower MI concentrations were associated with greater clinical improvement. These findings further support the view that MI alterations are region-dependent and may reflect broader neurobiological processes, including glial remodeling and normalization of limbic dysfunction, rather than a uniform pattern across psychiatric conditions [49,50]. Furthermore, reduced myo-inositol levels in the ACC have also been associated with greater depressive symptom burden across diagnostic groups, including patients with schizophrenia spectrum disorders [50,51].

For a more structured overview, selected studies evaluating MI levels in psychiatric disorders are presented in Table 2. As illustrated by these data, the reported alterations in MI levels are heterogeneous and appear to depend on the clinical condition, the biological matrix or brain region examined, and the methodology applied.

## 5. Conclusions

Current evidence suggests a possible role of selected inositol stereoisomers in anxiety-related psychopathology, potentially linked to intracellular second-messenger signaling. However, the available data are limited, uneven across stereoisomers, and partly inconsistent. The strongest, although still insufficient, body of evidence concerns MI, whereas SCI and DCI remain poorly represented in anxiety-related research. While myo-inositol appears to be generally well tolerated in available studies, firm clinical conclusions cannot yet be drawn. Further well-designed translational and clinical studies are required to clarify dosing strategies, relevant target populations, and long-term therapeutic value.

## Figures and Tables

**Table 1:** Summary of experimental and clinical studies investigating inositols in anxiety- and mood-related outcomes [white background—clinical studies; grey background—preclinical studies].

Inositol Isomer	Model	Aim/Methods	Results	Conclusion	Year	Ref.
MI	Patients with panic disorder with or without agoraphobia	Double-blind, randomized, placebo-controlled crossover trial assessing the efficacy of MI (12 g/day) over 4 weeks in reducing panic and agoraphobia symptoms.	MI significantly reduced the frequency and severity of panic attacks and agoraphobia compared with placebo; side effects were minimal.	MI demonstrated clinically relevant anxiolytic effects in panic disorder with good tolerability, supporting its potential as a therapeutic option targeting intracellular second-messenger pathways.	1995	[22]
MI	Adults with DSM-III-R post-traumatic stress disorder (PTSD)	Double-blind, placebo-controlled crossover study evaluating MI (12 g/day for 4 weeks) versus placebo in patients with PTSD. Outcomes assessed using the Impact of Event Scale (intrusion and avoidance subscales) and standard anxiety and depression rating scales.	MI did not produce significant improvements in core PTSD symptoms, including intrusion and avoidance, compared with placebo. A modest reduction in depressive symptoms was observed in a small subsample, while anxiety measures did not differ between treatment phases.	MI was not effective in alleviating core PTSD symptoms in this heterogeneous clinical population, suggesting limited anxiolytic efficacy beyond panic-related outcomes, although potential mood-related effects cannot be excluded.	1996	[28]
MI	Adults with obsessive–compulsive disorder	Double-blind, placebo-controlled crossover study evaluating the efficacy of MI administered at 18 g/day for 6 weeks in patients with obsessive–compulsive disorder. Symptom severity was assessed using the Yale–Brown Obsessive Compulsive Scale.	MI treatment was associated with significantly lower obsessive–compulsive symptom severity compared with placebo, as measured by Yale–Brown Obsessive–Compulsive Scale (YBOCS) scores.	MI demonstrated therapeutic efficacy in OCD in this controlled trial.	1996	[40]
MI	Male Wistar rats; elevated plus maze (EPM)	Experimental study assessing whether acute peripheral MI administration (2.5 g/kg in 10% saline solution, intraperitoneal) modulates anxiety-related behavior and blocks the anxiogenic effect of cholecystokinin tetrapeptide (CCK-4). Anxiety was evaluated using the elevated plus maze following CCK-4 or saline challenge.	CCK-4 significantly increased anxiety-like behavior, as expected. Acute MI administration also unexpectedly increased anxiety-related behavior and did not attenuate the anxiogenic effects of CCK-4. No significant changes were observed in total arm entries, suggesting effects were not due to altered locomotion.	Acute peripheral MI administration exerted anxiogenic-like effects in this model and failed to block CCK-induced anxiety, indicating that MI’s behavioral effects are strongly dependent on dosing regimen and timing, and may differ between acute and chronic exposure.		[26]
MI, L-Chiro-Inositol	Adult male Sprague–Dawley rats; EPM	The study investigated acute, chronic, and central effects of myo-inositol on anxiety-related behavior using the elevated plus maze. Rats received intraperitoneal myo-inositol across a wide dose range (acute dose–response), repeated daily intraperitoneal administration for 14 days, or intracerebroventricular myo-inositol injections. Behavioral assessment included time spent in open versus closed arms, number of arm entries, and central platform activity, with observers blinded to treatment conditions.	Acute intraperitoneal MI decreased time spent in open arms and increased closed-arm exploration, indicating anxiogenic-like effects comparable to a known anxiogenic agent. In contrast, chronic intraperitoneal administration reversed this pattern, increasing open-arm exploration and reducing closed-arm time, consistent with anxiolytic-like effects. Intracerebroventricular MI produced anxiolytic-like behavior, whereas L-chiro-inositol had no effect.	Inositol exerts dose-, route-, and duration-dependent effects on anxiety-related behavior, with chronic and central administration producing anxiolytic-like effects. These findings support a central, stereospecific mechanism and suggest that modulation of intracellular signaling pathways may underlie the behavioral effects of myo-inositol.	1997	[27]
MI, EI	Adult male Sprague–Dawley rats; EPM	Preclinical study examining the effects of chronic inositol administration on anxiety-like behavior. Rats received 11 consecutive daily intraperitoneal injections of MI, EI, or control solution. Anxiety-related behavior was assessed using the elevated plus-maze.	Both MI and EI reduced anxiety-like behavior compared with controls. Epi-inositol produced a more pronounced anxiolytic-like effect than MI.	Chronic inositol administration exerted anxiolytic-like effects in this animal model.	1998	[24]
MI (+L-chiro-inositol, EI)	Adult male Sprague–Dawley rats	Preclinical study investigating the effects of chronic dietary MI on locomotor behavior and regional brain MI concentrations, and comparing these effects with acute intracerebroventricular administration of MI and other inositol stereoisomers. Locomotion and rearing were measured using automated activity monitoring; regional brain MI levels were quantified by gas–liquid chromatography.	Chronic dietary MI significantly increased locomotor activity and rearing behavior and selectively elevated MI concentrations in the cerebral cortex and hippocampus, with no significant changes in the striatum or cerebellum. Acute intracerebroventricular administration of MI, L-chiro-inositol, or epi-inositol did not affect locomotor activity.	Chronic, but not acute, MI administration enhances locomotor activity and increases cortical and hippocampal MI levels, supporting a time- and region-dependent central effect of MI that may relate to its antidepressant-like properties observed in clinical studies.	1998	[41]
MI	DSM-IV obsessive–compulsive disorder (OCD) patients; add-on to SRI treatment	Double-blind, randomized, placebo-controlled crossover trial evaluating whether high-dose MI (18 g/day for 6 weeks) enhances clinical response in OCD patients with inadequate response to stable serotonin reuptake inhibitor therapy. Outcomes assessed using YBOCS, Hamilton Depression Scale, and Hamilton Anxiety Scale.	Both MI and placebo phases were associated with significant symptom improvement over time; however, no significant differences were observed between MI and placebo for obsessive–compulsive, anxiety, or depressive symptoms.	Adjunctive MI did not provide additional therapeutic benefit over placebo in SRI-treated, treatment-resistant OCD patients, suggesting limited efficacy of MI as add-on therapy in this population.	1999	[42]
MI	Adults with treatment-refractory obsessive–compulsive disorder	Open-label trial assessing the efficacy of MI augmentation (18 g/day for 6 weeks) added to ongoing serotonin reuptake inhibitor therapy in patients with OCD unresponsive to multiple prior SRI trials. Symptoms were evaluated biweekly using the YBOCS, Montgomery–Åsberg Depression Rating Scale, and Clinical Global Impressions Scale.	The majority of participants did not demonstrate clinically meaningful improvement with MI augmentation. A minority of patients showed a significant response in global improvement ratings.	MI augmentation did not produce a substantial benefit in most treatment-resistant OCD patients, although individual responders were observed, suggesting heterogeneity in response and the need for further mechanistic investigation.	1999	[43]
MI	Adult male Sprague–Dawley rats	Preclinical study evaluating the effects of chronic MI administration on depressive-like behavior. Rats received MI chronically (1.2 g/kg/day intraperitoneally or 10% dietary MI for 14 days). Behavioral outcomes were assessed using the Porsolt forced swim test and the reserpine-induced immobility model.	Chronic MI at 1.2 g/kg reduced immobility time and increased active struggling in the forced swim test. The same dosing regimen reduced complete immobility in the reserpine model without affecting general locomotor activity. Lower doses were ineffective. Oral dietary MI produced effects comparable to intraperitoneal administration.	Chronic MI administration exerted antidepressant-like effects in two distinct animal models, supporting a role for MI in modulating mood-related behaviors and providing a basis for further mechanistic and translational research.	1999	[25]
MI	Adults with panic disorder, with or without agoraphobia	Double-blind, controlled, random-order crossover trial comparing MI (up to 18 g/day) with fluvoxamine (up to 150 mg/day). Each treatment was administered for one month, and outcomes were assessed using the Hamilton Rating Scale for Anxiety, agoraphobia severity scales, and the Clinical Global Impressions Scale.	MI and fluvoxamine produced comparable improvements in anxiety severity, agoraphobia, and global clinical status. During the first treatment phase, MI was associated with a greater reduction in the weekly frequency of panic attacks than fluvoxamine. Gastrointestinal symptoms and fatigue were more frequently reported with fluvoxamine.	MI demonstrated efficacy comparable to an established SSRI in panic disorder, with a more favorable tolerability profile, supporting its potential as a well-tolerated alternative for patients sensitive to standard pharmacotherapy.	2001	[21]
Inositol/EI	Flinders Sensitive Line (FSL) rats model of depression and control adult male Sprague–Dawley rats	The study employed a 2 × 2 factorial design comparing rat strain (FSL vs. control) and treatment (inositol vs. placebo). Animals received daily inositol (1.2 g/kg) or placebo for 14 days, after which depressive-like behavior was assessed using a 5-min forced swim test, with quantified measures of immobility, swimming, and active struggling.	Inositol treatment attenuated the heightened immobility observed in FSL rats during the forced swim test, while leaving behavioral performance unchanged in control animals.	Anti-depressive potential	2002	[44]
Inositol	Adults with trichotillomania	Randomized, double-blind, placebo-controlled trial evaluating the efficacy and tolerability of inositol administered at doses of 6–18 g/day over 10 weeks. Symptom severity and clinical improvement were assessed using validated trichotillomania scales, global clinical ratings, and measures of anxiety, depression, and psychosocial functioning.	Inositol did not result in significantly greater reductions in trichotillomania symptoms compared with placebo on primary or secondary outcome measures. A numerically higher proportion of patients in the inositol group were rated as much or very much improved at study endpoint	Inositol did not demonstrate significant efficacy over placebo in trichotillomania at the group level, although a subset of individuals may derive clinical benefit, indicating possible heterogeneity in treatment response.	2017	[29]
MI	Pregnant women with low-risk singleton pregnancies	Double-blind, randomized controlled trial evaluating the effect of MI supplementation (2000 mg/day plus folic acid) administered for 10 weeks (gestational weeks 14–24) on sleep quality. Outcomes were assessed using the Pittsburgh Sleep Quality Index, with analyses adjusted for baseline covariates.	MI supplementation resulted in a statistically significant improvement in global sleep quality compared with placebo. Significant benefits were observed in subjective sleep quality, sleep duration, and habitual sleep efficiency.	MI supplementation during mid-pregnancy improved multiple dimensions of sleep quality, suggesting a beneficial effect on sleep-related central nervous system regulation.	2022	[33]
MI; combined with probiotics and micronutrients	Women planning conception and during pregnancy	Secondary analysis of a double-blind RCT (NiPPeR) assessing whether supplementation with MI combined with probiotics and enriched micronutrients from preconception through pregnancy influences mood, anxiety, and mental health functioning. Outcomes included EPDS, STAI-state, and SF-12v2 mental component scores assessed from preconception to 6 months postpartum.	No significant differences were observed between intervention and control groups in mood or anxiety scores during pregnancy or postpartum. The intervention group showed a modest but statistically significant improvement in overall mental health functioning from preconception to 6 months postpartum.	MI-based supplementation during preconception and pregnancy did not reduce anxiety or depressive symptoms but was associated with a small improvement in general mental health functioning postpartum, suggesting indirect or non-specific central nervous system effects.	2024	[36]
MI; combined with probiotics and trace elements	Pregnant women with mild to moderate perinatal depression	Retrospective cohort study evaluating the effects of combined MI, probiotic, and trace element supplementation initiated 3 months preconception and continued throughout pregnancy. Mood, anxiety, and quality of life were assessed preconception and postpartum using validated psychiatric scales; fetal growth parameters and neonatal outcomes were also recorded. Dose of MI 4 g/day.	The supplementation group showed lower postpartum anxiety and depressive symptom scores and improved quality of life compared with controls. Significant reductions in gestational diabetes and gestational hypertension were observed, alongside improved fetal growth parameters and reduced neonatal unit admissions.	Combined MI-based supplementation from preconception through pregnancy was associated with improved maternal mood, reduced anxiety, and more favorable pregnancy and neonatal outcomes	2025	[23]

**Table 2:** Overview of selected studies on alterations in inositol levels in psychiatric disorders [↑—increased, ↓—decreased, ^1^H-MRS—Proton Magnetic Resonance Spectroscopy, ACC—Anterior Cingulate Cortex, BDP—Bipolar Disorder, CSF—Cerebrospinal Fluid, Cr—Creatine, DLPFC—Dorsolateral Prefrontal Cortex, ECT—Electroconvulsive Therapy, MDD—Major Depressive Disorder, MI—Myo-inositol, OCD—Obsessive–compulsive disorder].

Disorder	Ongoing Treatment	Technique/Sample Type	Inositol Level	Year	Ref.
Primary affective disorder	Not specified	CSF	↓ MI level	1978	[52]
Manic BPD I	Lithium	^1^H-MRS	↑ MI in basal ganglia unchanged MI level in the occipital lobe	1992	[53]
Manic BPD I	Lithium	^1^H-MRS	No significant difference	1993	[54]
Social phobia	Drug-free	^1^H-MRS	↑ MI in grey matter	1997	[55]
MDD and depressed BPD I	Anti-depressant	^1^H-MRS	↓ MI in right frontal lobe	1998	[56]
Healthy subjects	Dietary MI—12 g/day for 8 days	^1^H-MRS	↑ (≈120%) in occipital gray matter after 4 days; returned toward baseline by day 8; no significant change in white matter	1999	[57]
MDD, BPD, schizophrenia	-	Postmortem brain tissue	No significant difference	2000	[58]
Manic BPD I, children	Drug-free	^1^H-MRS	↑ MI during mania vs. controls, ↓ MI after acute lithium treatment	2001	[59]
MDD	Drug-free	^1^H-MRS	↑ MI/Cr in frontal white matter; no significant gray matter difference	2002	[60]
Manic and mixed BPD I	Antipsychotic drugs/mood stabilizers	^1^H-MRS	No significant difference	2002	[61]
MDD	Drug-free	^1^H-MRS	↓ MI/Cr ratio	2003	[62]
BPD I	Lithium/valproate	^1^H-MRS	↑ MI in grey matter in lithium treated patients unchanged MI level in valproate treated patients	2004	[63]
Chronic alcoholism	Not specified	CSF	↑ SCI, highest in subjects with symptomatic encephalopathy	2004	[64]
Generalized social anxiety	Drug-free	^1^H-MRS	No significant difference	2005	[65]
MDD + migraine	Not specified	^1^H-MRS	↑ MI/tCr in bilateral DLPFC	2014	[66]
Schizophrenia spectrum disorder	Antipsychotic medication or drug-free	^1^H-MRS	↓ MI in ACC was associated with a greater depressive symptom burden	2015	[51]
OCD	Not specified	^1^H-MRS	↓ MI in medial prefrontal cortex	2015	[67]
Depression	ECT	^1^H-MRS	↑ MI in dorsomedial ACC during ECT; ↓ left hippocampal MI associated with symptom improvement	2016	[49]
Depression, sleep symptoms	Drug-free	^1^H-MRS	↓ MI in ACC and dorsolateral prefrontal cortex (DLPFC); negatively correlated with depression severity and anxiety	2017	[68]
Schizophrenia	Not specified	Systematic review and meta-analysis of ^1^H-MRS studies	↓ MI in medial prefrontal cortex	2018	[69]
Generalized anxiety symptoms associated with traffic-related air pollution exposure	Not specified	^1^H-MRS	↑ MI levels were associated with recent high TRAP exposure and greater generalized anxiety symptoms	2019	[46]
MDD, BDP	Not specified	^1^H-MRS	No significant difference	2024	[70]
Adolescent bipolar depression	One subject on lithium	^1^H-MRS	No significant difference in ventromedial prefrontal cortex	2025	[71]

## Data Availability

Further inquiries can be directed to the corresponding author.

## Abbreviations

The following abbreviations are used in this manuscript:

^1^H-MRS	Proton Magnetic Resonance Spectroscopy
ACC	Anterior Cingulate Cortex
BPD I	Bipolar Disorder type I
CSF	Cerebrospinal Fluid
Cr	Creatine
DCI	D-chiro-inositol
DLPFC	Dorsolateral Prefrontal Cortex
ECT	Electroconvulsive Therapy
EP	Epi-inositol
EPM	Elevated Plus Maze
FSL	Flinders Sensitive Line
MDD	Major Depressive Disorder
MI	Myo-inositol
OCD	Obsessive–compulsive Disorder
PMDD	Premenstrual Dysphoric Disorder
RCT	Randomized Controlled Trial
SCI	Scyllo-inositol
YBOCS	Yale–Brown Obsessive–Compulsive Scale
